# ﻿*Glossolomamagenticristatum* (Gesneriaceae), a new species from the Cordillera Oriental of the Colombian Andes

**DOI:** 10.3897/phytokeys.218.97590

**Published:** 2023-01-10

**Authors:** David Hoyos, Laura Clavijo, John L. Clark

**Affiliations:** 1 Grupo de Investigación en Recursos Naturales Amazónicos - GRAM, Facultad de Ingenierías y Ciencias Básicas, Instituto Tecnológico del Putumayo - ITP, Mocoa, Putumayo, Colombia Facultad de Ingenierías y Ciencias Básicas, Instituto Tecnológico del Putumayo Putumayo Colombia; 2 Herbario Etnobotánico del Piedemonte Andino Amazónico HEAA, Instituto Tecnológico del Putumayo - ITP, Cra. 17 14-85 (Corpoamazonia), Mocoa, Putumayo, Colombia Herbario Etnobotánico del Piedemonte Andino Amazónico, Instituto Tecnológico del Putumayo Putumayo Colombia; 3 Instituto Multidisciplinario de Biología Vegetal (IMBIV), CONICET-UNC, Universidad Nacional de Córdoba, CC 495, 5000, Córdoba, Argentina Universidad Nacional de Córdoba Córdoba Argentina; 4 Universidad Nacional de Colombia –Sede Bogotá, Facultad de Ciencias, Instituto de Ciencias Naturales, Bogotá, D.C., 111321, Colombia Universidad Nacional de Colombia Bogotá Colombia; 5 Marie Selby Botanical Gardens, 1534 Mound St., Sarasota, FL 34236, USA Marie Selby Botanical Gardens Sarasota United States of America; 6 Science Department, The Lawrenceville School, Lawrenceville, NJ 08648, USA The Lawrenceville School Lawrenceville United States of America

**Keywords:** Andes, biodiversity, Caquetá, *Glossoloma*, Huila, taxonomy

## Abstract

A narrowly endemic new species of *Glossoloma* is described from the Cordillera Oriental of the northern Andes, in the Colombian departments of Caquetá and Huila. *Glossolomamagenticristatum* J.L.Clark, D.Hoyos & Clavijo, **sp. nov.** differs from most other congeners by a habit that is usually epiphytic with elongate scandent subwoody shoots, the presence of a magenta corolla tube, and a creased calyx formed by tightly appressed adjacent lobes. A brief summary of Gesneriaceae diversity in the Colombia departments of Caquetá and Huila is discussed with an emphasis on the old highway between Florencia and Guadalupe. The conservation status of *G.magenticristatum* is assessed as Endangered (EN) based on IUCN Criteria.

## Abstract (es)

Se describe una especie de *Glossoloma* endémica de la Cordillera Oriental de los Andes del norte, en los departamentos de Caquetá y Huila. *Glossolomamagenticristatum* J.L.Clark, D.Hoyos & Clavijo, **sp. nov.** se diferencia de otras especies en el género por el hábito usualmente epífito con ramas alargadas, escandentes y subleñosas, el tubo de la corola magenta, y un cáliz con los lóbulos estrechamente adpresos y con margen ondulada. Se presenta una breve síntesis de la diversidad de Gesneriaceae en los departamentos de Caquetá y Huila, con un énfasis en la vieja carretera entre Florencia y Guadalupe. Se evalúa el estado de conservación de *G.magenticristatum* y se propone que se debería categorizar como En Peligro (EN), de acuerdo a los criterios de la UICN.

## ﻿Introduction

The flowering plant family Gesneriaceae is in the order Lamiales and comprises more than 3400 species and 150+ genera (Weber 2004; Weber et al. 2013). Gesneriaceae is a strongly supported monophyletic family classified into three subfamilies, seven tribes, and nine subtribes (Ogutcen et al. 2021). *Glossoloma* Hanst. is a member of the subtribe Columneinae, the largest clade in the subfamily Gesnerioideae with more than 560 species represented in 28 genera (Weber et al. 2020). The Columneinae comprises roughly 21% of all Gesneriaceae. *Glossoloma* is the sixth most diverse genus (30 spp.) in the Columneinae, after *Columnea* L. (200+ spp.), *Drymonia* Mart. (100+ spp.), *Nautilocalyx* Hanst. (60+ spp.), *Trichodrymonia* Oerst. (50+ spp.) and *Nematanthus* Schrad. (30+ spp.).

Hanstein (1854) initially recognized *Glossoloma* as a genus but later (Hanstein 1865) lumped it with four other genera into *Alloplectus* Mart. The non-monophyly of *Alloplectus* was recognized through phylogenetic studies (Clark and Zimmer 2003; Clark et al. 2006) and resulted in a reclassification of several genera, including most of the currently recognized species in *Glossoloma* (Clark 2005). *Glossoloma* is differentiated from other genera in the Gesneriaceae by the presence of resupinate flowers, a character not mentioned by Hanstein (1854, 1865), but more recently defined as a morphological synapomorphy for the genus (Clark and Zimmer 2003; Clark 2005; Clark et al. 2006). Other characters that define *Glossoloma* include a terrestrial habit of unbranched subshrubs and elongate tubular corollas that are ampliate apically and appear laterally compressed (Clark 2009).

*Glossoloma* ranges from Central America (southern Mexico) to South America (Bolivia). The center of diversity for *Glossoloma* is the western lowland Andean forests of Colombia and Ecuador, where more than 16 species occur. The type locality for *G.magenticristatum* is above 2000 meters on the Cordillera Oriental of the Colombian Andes. This region is characterized by abundant precipitation and high humidity (Ruíz-Hernández et al. 2021), and shares similar climatic conditions with the Cordillera Occidental. The Cordillera Occidental (western Andes) and Cordillera Oriental (eastern Andes) harbor more species of Gesneriaceae relative to the drier inter-Andean valleys (Van der Hammen 2000). For example, Clavijo et al. (2016) cite 66 species of Gesneriaceae in the department of Caquetá. In a forest near the type locality, Alvarez et al. (2019) recorded 21 species of Gesneriaceae, including four new records to the flora of Caquetá.

The type locality of *G.magenticristatum* is along the old highway between Florencia and Guadalupe, an area that is the type locality for several plant discoveries. Examples of species published from this region include *Fuchsiacuatrecasasii* Munz (Munz 1943), *Guzmaniacuatrecasasii* L.B.Sm. (Smith 1971), Juanulloaspeciosavarglabra Cuatrec. (Cuatrecasas 1958), *Kohlerialongicalyx* L.P.Kvist & L.E.Skog (Kvist and Skog 1992), *Piperresinaense* W.Trujillo (Trujillo and Jaramillo 2021), and *Pipertarquiense* W.Trujillo (Trujillo and Jaramillo 2021).

## ﻿Taxonomic treatment

### ﻿ Glossoloma magenticristatum

Taxon classificationPlantaeLamialesGesneriaceae

J.L.Clark, D.Hoyos & Clavijo sp. nov.

2983B119-7436-5806-BFE0-523283C2FE93

urn:lsid:ipni.org:names:77311680-1

Figs 1 , 2

#### Diagnosis.

Differs from all other *Glossoloma* by the presence of a magenta corolla tube and a creased calyx formed by tightly appressed adjacent lobes.

#### Type.

**Colombia. Caquetá**: Florencia, antigua vía Florencia–Guadalupe, hacía Cerro de Gabinete, 1°51'50"N, 75°40'22"W, 2387 m, 28 June 2021, *D. Hoyos, D. Sanín, A. Pérez & J. Castañeda 765* (holotype: COL; isotypes: COAH, CUVC, HEAA, HUA, HUAZ).

#### Description.

Terrestrial or epiphytic subshrub, branched, with elongate, scandent or horizontal shoots, to 2 m long, 4–8 mm in diameter. **Stems** subwoody, subquadrangular in cross-section, glabrescent proximally, velutinous distally, internodes 1–7 cm long. **Leaves** opposite, decussate, equal to subequal, coriaceous, enations present at the base of petioles, petioles 1–7.3 cm long, velutinous, subterete (flattened adaxially and rounded abaxially) in cross-section, reddish; blade elliptic to ovate, 8–15 × 3–7 cm, base cuneate to obtuse, apex attenuate, margin serrulate, adaxially green, puberulous, trichomes with swollen bases, abaxially light green suffused with pink, drying ferruginous with light brown venation, papillate, puberulent, primary vein velutinous, reddish proximally, lateral veins 6–9 per side, occasionally reddish, more pilose than blade. **Inflorescence** a reduced pair-flowered axillary cyme, with 1–8 flowers per node; peduncles absent or highly reduced (< 2 mm); bracts lanceolate to oblanceolate, 6.2–14.6 × 1.9–4.7 mm, dark purple, the apex acuminate to obtuse, sparsely pilose. **Flowers** resupinate; pedicels 1.2–4.0 cm long, dark vinaceous, velutinous, enations present (more abundant distally). **Calyx** with 5 lobes fused basally, lobes conduplicate with each lobe appressed to adjacent lobe and folded lengthwise with the margins curved outwards and forming a crease, light magenta, dark purple toward the middle and the base, subequal in size and shape, dorsal lobe slightly smaller, 1.4–2.0 × 0.5–1.2 cm, broadly ovate, base truncate, apex acute, margin repand to sinuate, pubescent on both surfaces, more densely pubescent toward the middle. **Corolla** zygomorphic, tubular, 3.8–4.3 cm long, gibbous basally on lower surface, spur absent, tube broadly widened on dorsal surface (not widened on ventral surface), long axis of corolla perpendicular relative to calyx; corolla tube light magenta outside, whitish with dark red to magenta splotches inside, 2.2–2.5 cm long, basal gibbosity 1.2–1.6 cm long, base 9–11 mm in diameter, middle widened, becoming apically ventricose on upper surface, throat slightly constricted, appearing laterally compressed, 4–6 mm in diameter, densely pilose with translucent white trichomes outside, mostly glabrous, with glandular trichomes apically in the inner surface of throat; limb 11–14 mm wide, with 5 reflexed lobes, lobes subequal, 3.2–5.3 × 4.1–6.4 mm, rotund, margin entire, cream yellow with dark red to magenta splotches proximally and light vinaceous splotches distally, glabrous on both surfaces. **Androecium** of 4 stamens; filaments connate at the base forming a filament curtain for 6–9 mm, free portion of filaments 1.5–1.9 cm long, glabrous; anthers rectangular, 2.9–3.4 × 2.7–2.8 mm, twice as wide during anthesis, dehiscing by longitudinal slits; staminode absent. **Gynoecium** with two connate glands, each nectary truncate and shallowly bilobed, 2–3 mm long, glabrous; ovary superior, densely pilose, 8–10 × 4–6 mm; style ca. 16 mm long, glabrous, stigma stomatomorphic. **Fruit** a bivalved ovoid fleshy capsule, laterally compressed, 12–15 × 13.0–14.1 mm, densely pilose, the valves white on both surfaces, valves reflexed to 180° when mature, revealing a central cone of fleshy white funicular tissue covered by an ephemeral thin brown pericarp. **Seeds** numerous, initially covered by the endocarp, but immersed in the central cone of funicular tissue, each seed 0.9–1.3 × 0.3–0.4 mm, dark brown, ellipsoid, and longitudinally striate.

#### Phenology.

Mature flowers were documented during June and September and immature floral buds during January. Immature fruits were documented during September and October, and mature fruits were observed in October.

#### Etymology.

The specific epithet is derived from two adjectives that reflect the unique characteristics of *Glossolomamagenticristatum.* The corolla is magenta, a color not found in other members of *Glossoloma*. The adjacent calyx lobes are tightly appressed and form a crease that appears winged or crested (Fig. 2B), which reflects the second part of the specific epithet, “cristate.”

**Figure 1.**
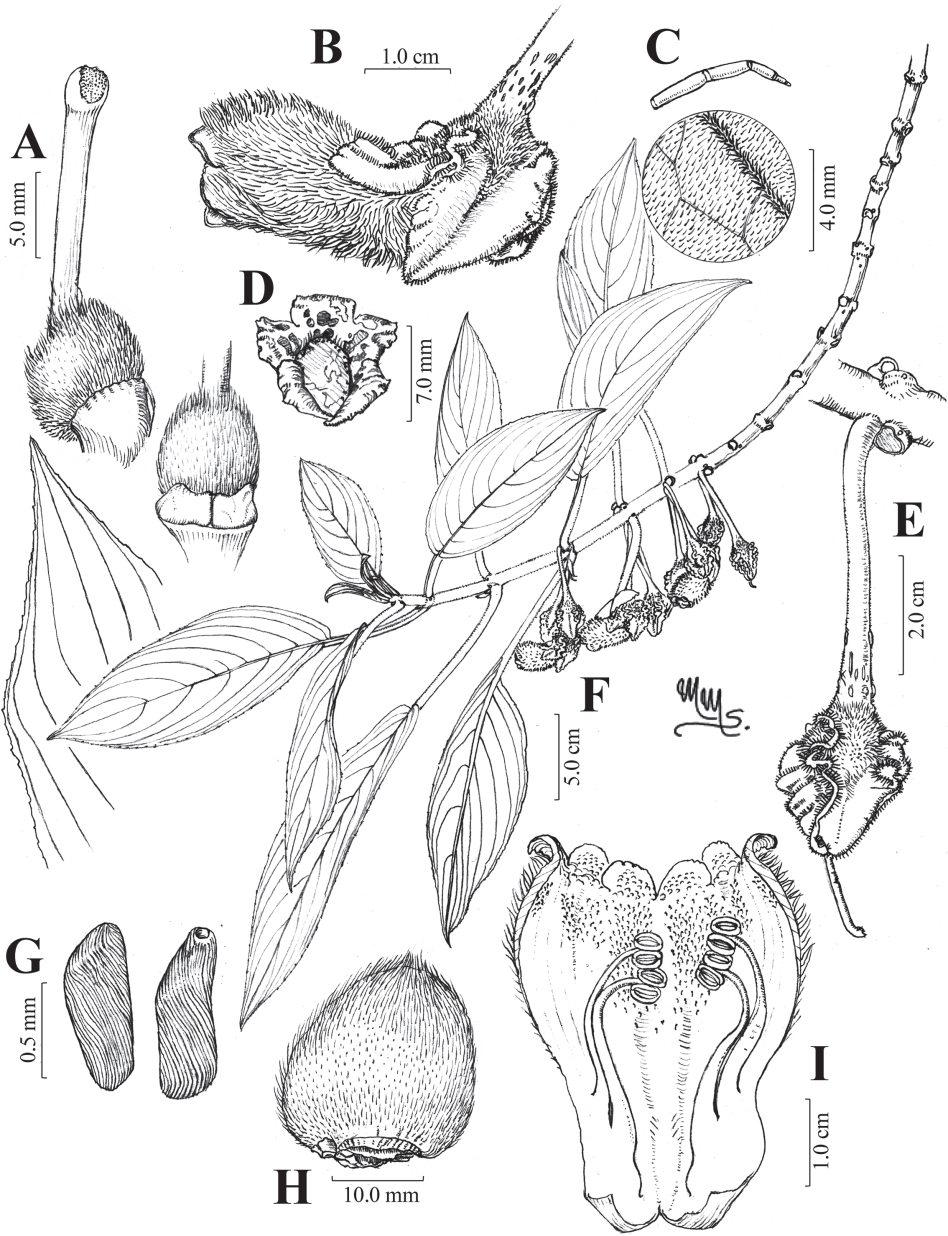
*Glossolomamagenticristatum* J.L.Clark, D.Hoyos & Clavijo **A** gynoecium featuring nectary of two connate glands **B** lateral view of mature flower **C** adaxial leaf surface with inset featuring multicelled trichome **D** face view of flower **E** gynoecium surrounded by cristate calyx lobes **F** habit **G** seeds **H** immature cone-shaped fruit **I** opened corolla featuring mature stamens. Illustration by *M. Morales* from *Hoyos et al. 765*.

**Figure 2.**
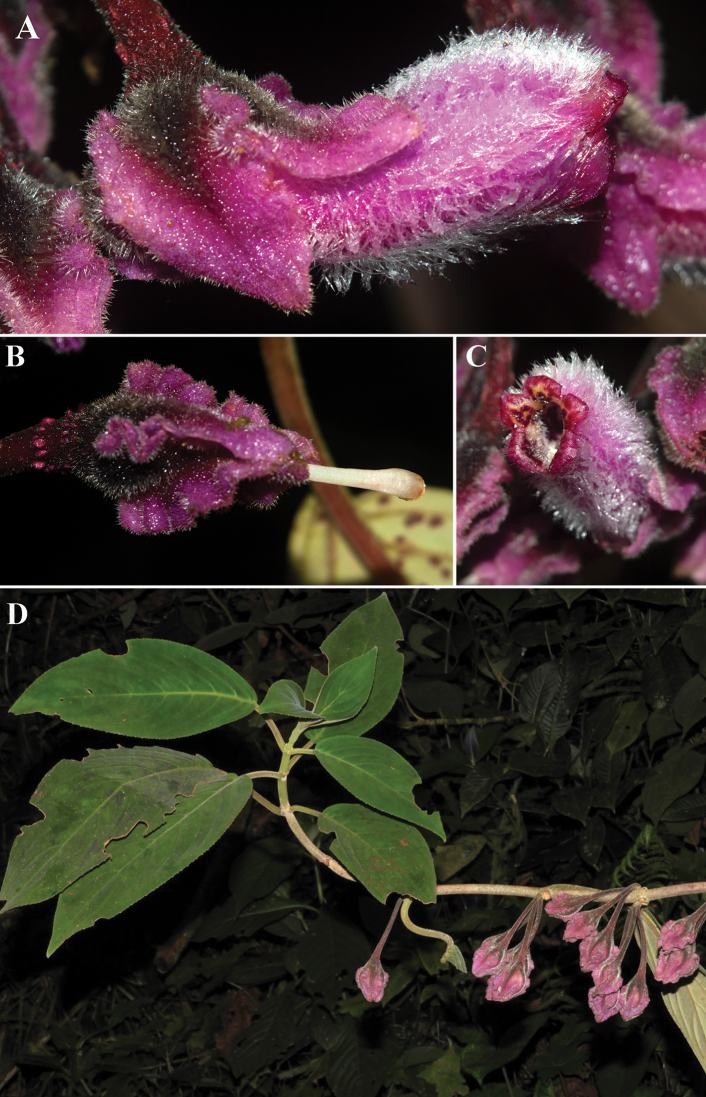
*Glossolomamagenticristatum* J.L.Clark, D.Hoyos & Clavijo **A** lateral view of mature flower **B** lateral view of calyx featuring cristate lobes **C** front view of flower **D** habit (**A, B, C***Hoyos et al 765***D***Hoyos & Castañeda 233*). Photos **A, B, C** by D. Sanín and photo **D** by D. Hoyos.

#### Distribution and preliminary assessment of conservation status.

*Glossolomamagenticristatum* is endemic to the Colombian Cordillera Oriental (Eastern Cordillera) of the northern Andes, between 1900 and 2400 m elevation (Fig. 4). The three known populations of *Glossolomamagenticristatum* were documented growing on roadsides, characterized by shaded secondary forest. Two of the three known populations are documented with collections. A third population in the Huila department (3°19'3.96"N, 74°39'42.32"W) is based on an observation and photograph from April of 2019 on iNaturalist by Jorge Luis Peña. Following the IUCN Red List Categories and Criteria (IUCN 2022) and guidelines of the IUCN Standards and Petitions Committee (IUCN 2022), *Glossolomamagenticristatum* is categorized as Endangered (EN) based on the following criteria: B1ab (III) + 2ab (III), extent of occurrence (EOO) is calculated at 378.997 km^2^ (criterion B1 < 5000 km^2^), and area of occupancy (AOO) is calculated at 12 km^2^ (criterion B2 < 500 km^2^). The population from the type locality is at risk from periodic disturbance due to the removal of roadside vegetation by maintenance staff on the Florencia–Guadalupe road and globally by the ongoing decline of Andean forests from colonization and agriculture.

#### Comments.

**Figure 3.**
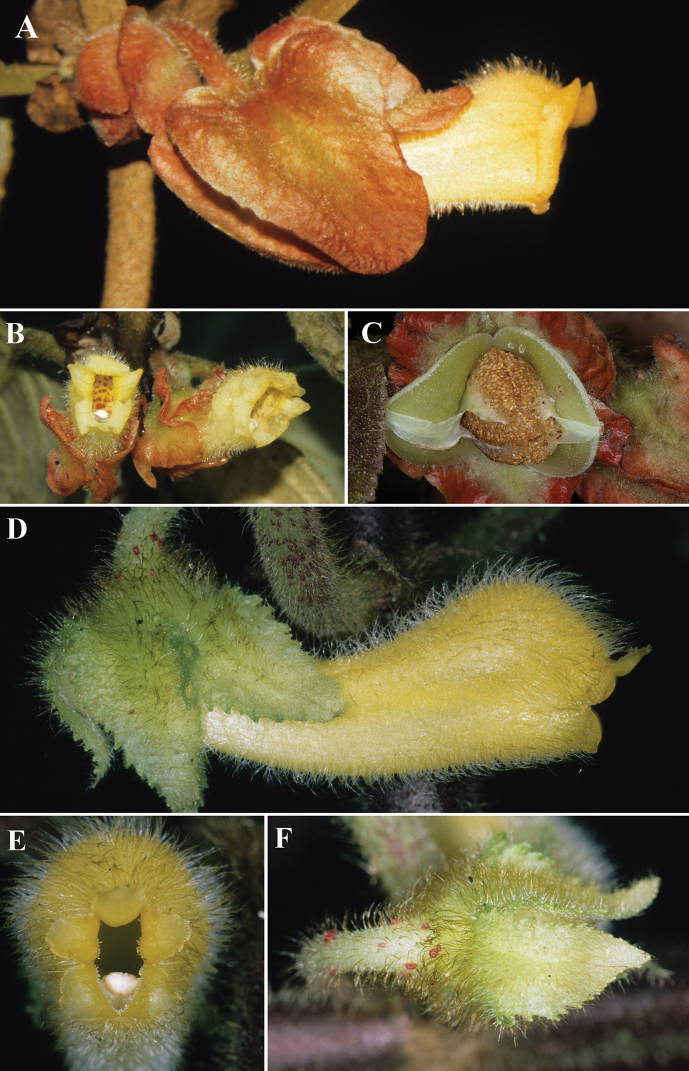
*Glossolomaserpens* J.L.Clark & L.E.Skog (J.L.Clark) and *G.chrysanthum* (Planch. & Linden) J.L. Clark **A** lateral view of mature flower of *G.serpens***B** front view of mature flower of *G.serpens***C** mature fruit of *G.serpens***D** lateral view of mature flower for *G.chrysanthum***E** front view of mature flower of *G.chrysanthum***F** calyx of *G.chrysanthum* (**A***J.L.Clark 5627***B***J.L.Clark 5996***C***J.L.Clark 9834***D, E, F***J.L.Clark 6872*). All photos by J.L. Clark.

**Figure 4.**
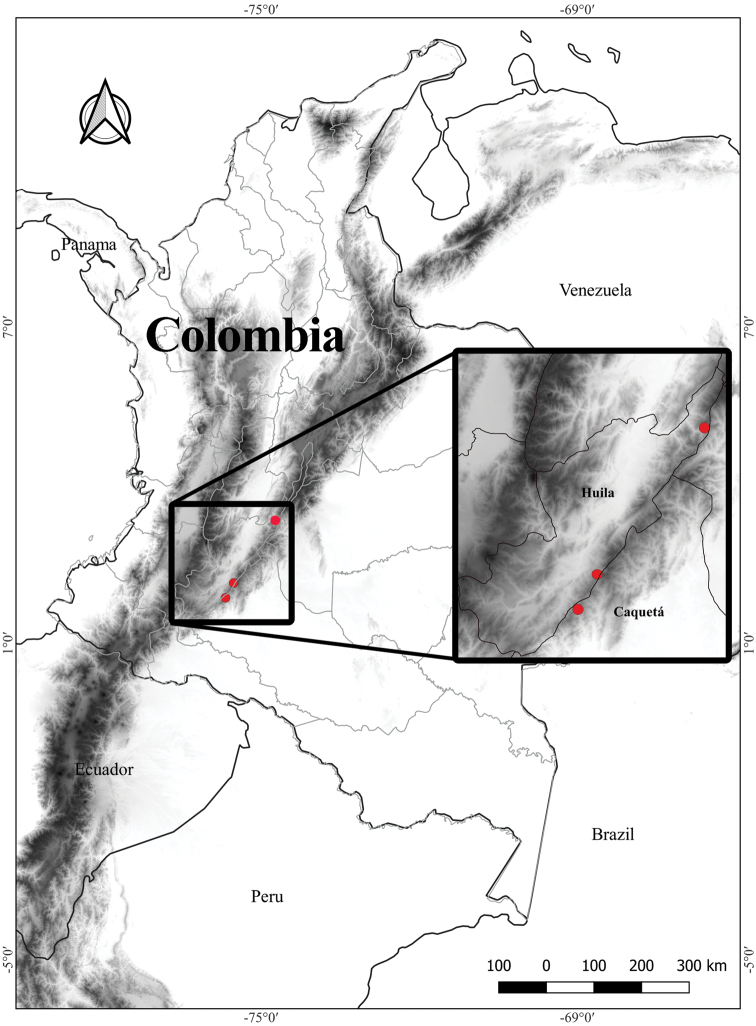
Distribution map of *Glossolomamagenticristatum* J.L.Clark, D.Hoyos & Clavijo in Colombia with inset featuring collection localities in the departments of Caquetá and Huila.

Most species of *Glossoloma* share a habit defined as unbranched terrestrial subshrubs. The presence of an epiphytic habit is rare, and it is even more unusual for epiphytic *Glossoloma* to have elongate climbing shoots. Currently known species of *Glossoloma* with an epiphytic habit and elongate shoots include *G.chrysanthum* (Planch. & Linden) J.L.Clark, *G.penduliflorum* (M.Frieberg) J.L.Clark, *G.scandens* J.L.Clark, and *G.wiehleri* J.L.Clark & F.Tobar. The description here of *G.magenticristatum* brings the total number of epiphytic *Glossoloma* with elongate shoots to five species. *Glossolomamagenticristatum* is most similar to *G.serpens* (Fig. 3), but readily distinguished by the presence of a magenta corolla (Fig. 2) (vs. red to yellow corolla in *G.serpens*, Fig. 3A), ovate calyx lobes (vs. broadly ovate in *G.serpens*, Fig. 3A), and coriaceous leaves (vs. papyraceous in *G.serpens*). The presence of an epiphytic habit with elongate shoots is also found in *G.chrysanthum* from Venezuela. These two species are geographically isolated, with *G.magenticristatum* endemic to Colombia (Fig. 4) and *G.chrysanthum* endemic to Venezuela. In addition, these species differ by a corolla length 3.8–4.3 cm in *G.magenticristatum* (vs. corolla length to 3.5 cm in *G.chrysanthum*), calyx magenta in *G.magenticristatum* (vs. light green calyx in *G.chrysanthum*, Fig. 3F), and magenta corolla in *G.magenticristatum* (vs. uniformly yellow corolla in *G.chrysanthum*, Fig. 3D).

#### Additional specimens examined.

**Colombia. Caquetá**: Florencia, Cerro de Gabinete, Antigua vía Florencia–Guadalupe, 1°52'51.5"N, 75°4'46.5"W, 2000 m, 15 Sep 2018, *D. Hoyos & M. Cuellar 103* (COL); Florencia, Cerro de Gabinete, Antigua vía Florencia–Guadalupe, 1°52'51.5"N, 75°4'46.5"W, 2000 m, 22 Sep 2019, *D. Hoyos & J. Castañeda 233* (HUAZ, HEAA). **Huila**: Garzón, vereda Las Mercedes, borde de bosque secundario, 2°8'44.5"N, 75°31'9.6"W, 1960 m, 27 Dec 2021, *J.L. Peña, E. Rojas & D. Hoyos 924* (HEAA, HUAZ).

## Supplementary Material

XML Treatment for Glossoloma magenticristatum
